# In vivo analysis of iridocorneal angle parameters with spectral-domain optical coherence tomography in children with Neurofibromatosis type 1

**DOI:** 10.1038/s41433-025-03840-z

**Published:** 2025-05-14

**Authors:** Murat Gunay, Ibrahim Mert Kurt, Ugur Yilmaz, Adem Turk, Dilek Uzlu, Busra Kose, Pinar Ozkan Kart, Ali Cansu

**Affiliations:** 1 Karadeniz Technical University, Faculty of Medicine, Department of Ophthalmology, Trabzon, Turkey; 2 Trabzon Of State Hospital, Department of Ophthalmology, Trabzon, Turkey; 3 University of Health Sciences, Trabzon Kanuni Training and Research Hospital, Department of Pediatric Neurology, Trabzon, Turkey; 4 Karadeniz Technical University, Faculty of Medicine, Department of Pediatric Neurology, Trabzon, Turkey

**Keywords:** Outcomes research, Eye manifestations

## Abstract

**Purpose:**

To evaluate iridocorneal angle (ICA) parameter measurements with spectral-domain optical coherence tomography (SD-OCT) in children with Neurofibromatosis type 1 (NF1) and to compare them with those in healthy children.

**Methods:**

Twenty children with NF1 and 33 age- and sex-matched healthy controls were enroled. All subjects underwent ICA imaging with SD-OCT. Schlemm canal diameter (SCD), anterior chamber angle (ACA), angle opening distance (AOD500 and AOD750), trabecular-iris space area (TISA500 and TISA750) and scleral spur length (SSL) were examined in the temporal sections and measured with customized software. Also, iris thickness (IT) was measured as the distances between the anterior and posterior iris surface, at 1 mm (IT-1), 2 mm (IT-2) and 3 mm (IT-3) from the edge of the pupil.

**Results:**

Mean ACD was significantly narrower in NF1 group (*p* = 0.003). Mean levels of SCD (*p* < 0.001), ACA (*p* = 0.001), AOD500 (*p* = 0.001), AOD750 (*p* < 0.001), TISA500 (*p* < 0.001) and TISA750 (*p* < 0.001) were significantly lower in NF1 group. Mean SSL-1 (*p* = 0.19) and SSL-2 (*p* = 0.56) measurements were found to be similar between the groups. Mean IT-1 (*p* < 0.001), IT-2 (*p* < 0.001) and IT-3 (0.03) were observed to be significantly higher in NF1 group. There was significant correlation between ACD and IT. Also, ACD and IT showed significant associations with the ICA parameters.

**Conclusion:**

There was a significantly narrower ICA morphology in NF1 children. SCD was significantly decreased in children with NF1. A significantly thicker iris in NF1 children may offer a possible impact of iris morphology on narrower ICA parameters and SCD.

## Introduction

Neurofibromatosis type 1 (NF1) is a rare autosomal dominant neurogenetic disorder caused by heterozygous mutations of the NF1 gene on chromosome 17q11.2 with a prevalence of 1 in 2500 to 3000 individuals [1]. The disease is typically characterized by café-au-lait spots, dermal or plexiform neurofibromas, skeletal dysplasia and axillary freckling. Most common ocular findings of NF1 are iris hamartomas, so called Lisch nodules. Other ocular manifestations include subcutaneous eyelid and periocular neurofibromas, choroidal hamartomas, optic pathway gliomas, choroidal nevi and retinal astrocytomas [2].

Glaucoma has been reported as a rare manifestation of NF1 often diagnosed in childhood and adolescence. Several pathophysiologic mechanisms have been suggested for glaucoma development in NF1 which include infiltration of iridocorneal angle (ICA) by neurofibromas, synechial angle closure as a result of fibrovascular process and developmental abnormalities in the ICA [3]. Investigators have examined ICA with gonioscopy and demonstrated some characteristic signs indicating underdevelopment of the ICA in NF1 patients under 18 years [4]. However, gonioscopic examination is not always easy in the paediatric population.

Anterior segment optical coherence tomography (AS-OCT) can provide in vivo non-contact qualitative and quantitative evaluation of the anterior chamber in a non-invasive way [5]. During recent years, AS-OCT has gained popularity to identify structural alterations in various ophthalmic conditions [6–8].

To the best of our knowledge, no in vivo assessment of the ICA has been made in paediatric NF1 patients with OCT. Therefore, in the present study, we aimed to investigate ICA changes in children with NF1 and to compare the results with those of healthy children. For that purpose, spectral domain OCT (SD-OCT) was used to perform measurements of Schlemm’s canal diameter (SCD), anterior chamber angle (ACA), angle opening distance (AOD), trabecular-iris space area (TISA), scleral spur length (SSL) along with iris thickness (IT).

## Materials and methods

In this prospective and cross-sectional study, 20 children with NF1 (NF1 group) and 33 healthy children without any ocular and/or systemic disease (control group) were enroled between June 2022 and February 2024 at a tertiary academic centre. A local ethical approval was obtained for the present study and the study followed the principles laid out in the Declaration of Helsinki. An informed consent was taken from the parents before the procedures. All children in NF1 group had already fulfilled the NF1 National Institute of Health criteria in Paediatric Neurology Department at the same institute during recruitment [9].

All participants in the study were required to be <17 years of age. All patients with NF1 and healthy participants underwent detailed ophthalmic examination, including refractive error measurement, best corrected visual acuity, slit-lamp biomicroscopic examination, stereoscopic fundoscopic evaluation and intraocular pressure (IOP) measurement. Axial length (AL), anterior chamber depth (ACD) and central corneal thickness (CCT) measurements were also obtained with an optical biometer device (AL-Scan; Nidek Co., Ltd., Japan).

### Eligibility criteria

Children with a diagnosis of NF1 were consecutively included in the study. Children with a history of any ocular surgery, intervention and/or trauma, any corneal disease, uveitis, dense media opacities, any systemic disease affecting ocular structures (i.e., diabetes mellitus, connective tissue disorder) were excluded. Also, children who could not cooperate with the OCT examination or those from whom poor imaging quality was obtained, or whose families are not willing to participate were excluded. Similar exclusion criteria were taken into consideration in the part of the study that constituted the healthy control group.

### Spectral-domain optical coherence tomography analysis

**Fig. 1:**
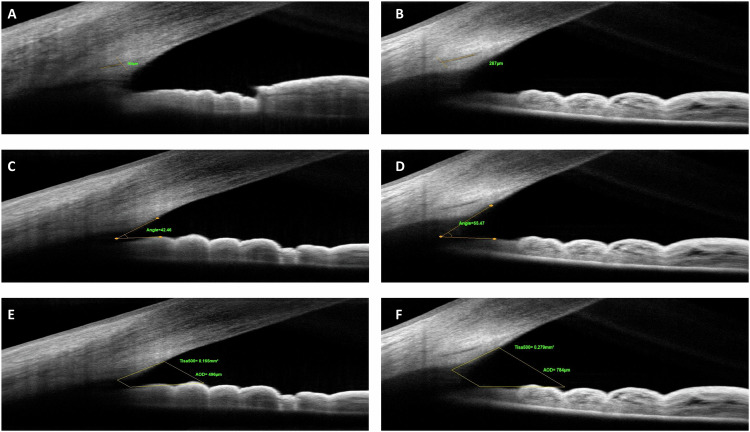
Examples regarding measurements of iridocorneal angle parameters with spectral-domain optical coherence tomography in NF1 and control cases in the study. Measurement of Schlemm canal diameter in NF1 (**A**) and control (**B**) case. Measurement of anterior chamber angle in NF1 (**C**) and control (**D**) case. Measurement of trabecular-iris space area and angle opening distance at 500 µm in NF1 (**E**) and control case (**F**).

**Fig. 2:**
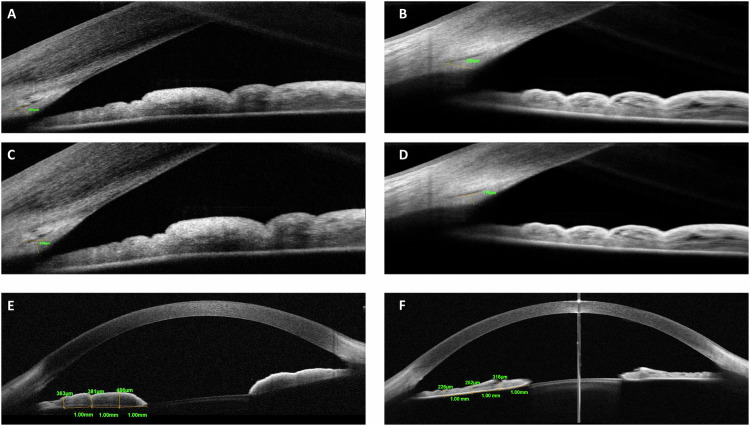
Examples regarding measurements of iridocorneal angle parameters with spectral-domain optical coherence tomography in NF1 and control cases in the study. Measurement of scleral spur length-1 in NF1 (**A**) and control (**B**) case. Measurement of scleral spur length-2 in NF1 (**C**) and control (**D**) case. Measurement of iris thickness 1 mm, 2 mm and 3 mm from the edge of the pupil in NF1 (**E**) and control case (**F**).

In an attempt to evaluate ICA parameters, all children in the study underwent imaging with a SD-OCT device (Solix full-range OCT, Optovue Inc., Freemont, CA, USA) with a scan speed of 120,000 A-scans per second, an 840 nm laser diode and a 5 µm axial resolution performed in the same dark room (mesopic) conditions. All the images in the study were obtained by an experienced technician with the patient sitting upright, keeping the eyes open as wide as possible. The temporal limbus was imaged after adjustment of the participant’s fixation to the nasal area. External fixation target of the device was also used to guide participant fixation. OCT scans were obtained using a 16-mm corneal lens adapter (cornea anterior module [CAM]), fixed in front of the ocular lens, in order to help to image the ICA with the software of the device set to Angle mode. In this mode, on a 3-mm line centred at the limbus, 32 B-scans each comprising 1024 A-scans were automatically obtained with a 0.04 second of duration for each B-scan. Images of a quality defined by a signal strength intensity above 30 were used for the final analysis. Measurements of all ICA parameters were made manually on these images using the software of the device based on the definitions in previous literature data [7, 10]. *SCD* was defined as the axial length of the oblong-elliptic, thin, lucent and hyporeflective space, generally located close to the trabecular meshwork outside and posterior to it. *ACA* was measured by tracing a line from the angle recess to the Schwalbe’s line and another line on the surface of the iris to the perpendicular point on the Schwalbe’s line. *AOD* and *TISA* were measured using the TISA/AOD Measurement Tool Angle Scan already set in the software of the device according to the manufacturer’s user manual. The number 500 or 750 is the distance (in µm) measured between the two upper points along the posterior cornea surface. One of these two upper points were located on the SS and the other one on the posterior cornea surface 500 or 750 μm away from SS. The *AOD* is the distance from cornea to iris. The area is measured as the trapezoidal area encompassed by these four points. Apart from these, *SSL* was also measured in two different previously reported methods [11]. In method 1 (*SSL-1*), the measurement was taken from the tip of the SS to the level of the posterior end of SC, along the anterior side of the SS [12]. In method 2 (*SSL-2*), the measurement was taken from the tip of the SS, directly to the level of the posterior end of SC [13]. *IT* was analysed with the software of the device set to Full range AC mode using a 18-mm CAM lens. *IT* was measured as the distances between the anterior and posterior iris surface, at 1 mm (IT-1), 2 mm (IT-2) and 3 mm (IT-3) from the edge of the pupil. Figures 1, 2 demonstrate representative measurements of the ICA parameters on SD-OCT in the study. One experienced observer (M.G.) took three consecutive measurements on the same SD-OCT images for each parameter at different measurement sessions, and the average of these measurements were used in the analysis. Furthermore, to determine intra-observer reproducibility in the study, the same consecutive measurements were also used.

### Statistical analysis

All data obtained from the participants were evaluated in the computer package programme SPSS 13.0.1 (SPSS, Chicago, IL; license number: 9069728, KTU Trabzon). Measurement data were presented as mean (standard deviation), and the conformity of these data to normal distribution was evaluated with the one-sample Kolmogorov-Smirnov test. Mann–Whitney U-test was used for comparisons owing to the low number of cases in the NF1 group. Categorical data were reported as number of cases and percentages, and comparisons were made using χ2 test. Pearson correlation test was also used to investigate the relationship between the measurement data of the entire study group. The reliability of the SD-OCT measurements was assessed by calculating intraclass correlation coefficients (ICC) for intra-observer agreement. The statistical significance level was accepted as *p* < 0.05. Post hoc power analysis using G*Power was also performed to estimate the power for several parameters (SCD, ACA and AOD500) in the study. The results of post hoc power analysis showed a power of 94%, 97% and 98% for SCD, ACA and AOD500, respectively.

## Results

Totally 62 children were considered for inclusion in the study; 3 in the NF1 group and 2 in the control group were excluded due to no identification of SC, and 2 in the NF1group and 2 in the control group were excluded due to lack of cooperation during OCT examinations. Finally, 53 children, 20 with NF1 and 33 healthy controls were included in the analysis. All children (100%) in NF1 group had café au lait spots, 17 (85%) had Lisch nodules, 4 (20%) had axillary freckling, 4 (20%) had cerebral hamartomatous lesions, 2 (10%) had plexiform neurofibroma not involving ocular structures, 2 (10%) had epilepsy, 1 (5%) had ectropion uvea and 1 (5%) had optic pathway glioma. No significant differences were observed between the two groups in terms of mean age, sex distribution, IOP, refraction, AL and CCT (*p* > 0.05 for all). The mean ACD was found to be significantly narrower in NF1 group than in control group (*p* = 0.003). Table 1 shows clinical characteristics of the study population.

**Table 1:** Clinical characteristics of the study population.

	NF 1 group *N* = 20	Control group *N* = 33	*p*
Age (years)	12.22 (3.11) (7–16)	11.12 (2.31) (7–16)	0.16
Sex, Female / male N (%)	7 (35) / 13 (65)	19 (57.6) / 14 (42.4)	0.11
IOP (mmHg)	15.21 (2.71) (11–20)	15.63 (2.62) (10–20)	0.51
Spherical refraction (dioptres)	0.15 (0.50) (–0.50 to 1.00)	–0.18 (0.91) (–2.00 to 1.75)	0.21
Cylindrical refraction (dioptres)	0.00 (0.57) (–0.75 to 1.00)	–0.02 (0.65) (–1.00 to 1.75)	0.71
AL (mm)	22.9 (0.9) (21.6–24.8)	23.1 (0.9) (21.3–24.7)	0.31
ACD (mm)	3.51 (0.19) (3.16–3.81)	3.74 (0.25) (3.27–4.25)	0.003
CCT (µm)	565.45 (21.15) (527–609)	562.31 (40.92) (456–686)	0.91

*p* < 0.05

*Values are presented as mean (standard deviation) and (minimum—maximum).

*NF* neurofibromatosis, *IOP* intraocular pressure, *AL* axial length, *ACD* anterior chamber depth, *CCT* central corneal thickness.

Table 2 shows measurement results of the ICA parameters in each group. Mean levels of SCD (*p* < 0.001), ACA (*p* = 0.001), AOD500 (*p* = 0.001), AOD750 (*p* < 0.001), TISA500 (*p* < 0.001) and TISA750 (*p* < 0.001) were found to be significantly lower in NF1 group compared to control group. Mean SSL-1 (*p* = 0.19) and SSL-2 (*p* = 0.56) values did not show significant differences between the groups. Mean levels of IT-1 (*p* < 0.001), IT-2 (*p* < 0.001) and IT-3 (0.03) measurements were observed to be significantly higher in NF1 group than in control group.

**Table 2:** Comparison of the iridocorneal angle parameters between the groups.

	NF1 group *N* = 20	Control group *N* = 33	*p*
SCD (µm)	253.41 (74.54) (154–420)	312.46 (51.19) (238–476)	<0.001
ACA (degrees)	35.06 (5.81) (28.32–48.55)	40.75 (5.65) (32.06–53.10)	0.001
AOD500 (µm)	440.25 (86.18) (308–638)	579.52 (154.54) (304–884)	0.001
AOD750 (µm)	606.05 (103.36) (458–922)	765.24 (157.66) (495–1070)	<0.001
TISA500 (mm^2^)	0.154 (0.029) (0.104–0.191)	0.198 (0.048) (0.110–0.292)	<0.001
TISA750 (mm^2^)	0.284 (0.049) (0.196–0.403)	0.372 (0.086) (0.227–0.518)	<0.001
SSL-1 (µm)	195.20 (21.14) (166–240)	186.76 (25.87) (139–241)	0.19
SSL-2 (µm)	159.75 (25.91) (115–206)	155.64 (23.59) (116–204)	0.56
IT-1 (µm)	416.35 (78.31) (286–586)	297.52 (63.72) (204–417)	<0.001
IT-2 (µm)	364.40 (78.62) (255–535)	266.09 (71.48) (117–451)	<0.001
IT-3 (µm)	309.85 (77.18) (216–488)	255.31 (56.72) (161–366)	0.03

*Values are presented as mean (standard deviation) and (minimum—maximum).

*NF* neurofibromatosis, *SCD* Schlemm canal diameter, *ACA* anterior chamber angle, *AOD* angle opening distance, *TISA* trabecular-iris space area, *SSL* scleral spur length, *IT* iris thickness.

*p* < 0.05.

A significant but weak correlation of ACD with IT-1 (r = –0.34; *p* = 0.01) and IT-2 (r = –0.29; *p* = 0.03) was observed. A statistically significant weak negative correlation was also recorded between SCD and IT-1 and IT-2 values. Furthermore, IT-1 and IT-2 exhibited a distinct negative linear relationship with ICA parameters (AOD500, AOD750, TISA500 and TISA750). Table 3 summarizes correlation analysis results obtained in the study.

**Table 3:** Correlation results obtained in the study.

	SCD	ACA	AOD500	AOD750	TISA500	TISA750
ACD	*r* = 0.16 *p* = 0.26	*r* = 0.45 *p* = 0.001	*r* = 0.38 *p* = 0.005	*r* = 0.34 *p* = 0.012	*r* = 0.39 *p* = 0.003	*r* = 0.36 *p* = 0.008
IT-1	*r* = –0.30 *p* = 0.027	*r* = –0.34 *p* = 0.012	*r* = –0.46 *p* < 0.001	*r* = –0.43 *p* = 0.001	*r* = –0.49 *p* < 0.001	*r* = –0.46 *p* = 0.001
IT-2	*r* = –0.27 *p* = 0.04	*r* = –0.24 *p* = 0.08	*r* = –0.44 *p* = 0.001	*r* = –0.43 *p* = 0.001	*r* = –0.37 *p* = 0.007	*r* = –0.43 *p* = 0.001
IT-3	*r* = –0.26 *p* = 0.06	*r* = –0.15 *p* = 0.29	*r* = –0.25 *p* = 0.08	*r* = –0.24 *p* = 0.07	*r* = –0.26 *p* = 0.06	*r* = –0.28 *p* = 0.04

*SCD* Schlemm canal diameter, *ACA* anterior chamber angle, *AOD* angle opening distance, *TISA* trabecular-iris space area, *ACD* anterior chamber depth, *IT* iris thickness.

*r* = Pearson correlation *p* < 0.05.

As for reproducibility, intra-observer agreement was very good for all ICA parameter measurements (Supplemental material).

## Discussion

To the best of our knowledge, this is the first in vivo study elaborating ICA morphology with SD-OCT in NF1 in a paediatric population. We observed significantly narrower ACA, AOD, TISA and increased IT values in NF1 cases when compared to controls. Also, we firstly exhibited significantly decreased SCD in NF1 children in the literature. Furthermore, baseline characteristics in the present study, including age, gender distribution, IOP, refractive errors and AL, did not differ between the two groups, hence the elimination of such confounders would result in more reliable comparisons of study outcomes between the groups.

There are several reports demonstrating narrower ICA parameters mainly based on lower AOD and decreased ACA values in adult NF1 patients [14, 15]. Duru et al. [15] have performed ocular biometric and ICA evaluation by using Scheimpflug camera system and found decreased ACD, narrower ACA and reduced anterior chamber volume in adult individuals with NF1. There is scarce literature data regarding assessment of ICA with OCT in NF1 patients. In a study, Duru et al. [14] have identified lower AOD values in adult patients with NF1 than in healthy subjects, as well as significantly narrower ACA and thicker IT values by using an AS-OCT system. Consistently, we observed significantly smaller ACD, narrower ICA and thicker iris morphology in NF1 children in our study. Interestingly, there was significant negative correlations between IT values and ICA parameters, suggesting that increased IT might be a potential contributor to decreased ACD and narrower ICA morphology at least in the present paediatric NF1 cohort. Previous studies on the same topic have considered melanocytic and fibroplastic proliferations on the anterior surface of the iris and NF-related thickening of the ciliary body as potential contributing factors on thickening of iris and narrowing of ICA [14–16]. Investigators have demonstrated several histopathological findings in uveal tissue of NF1 patients, including aggregation of melanocytes and stromal alterations on the anterior surface of the iris, consistent with Lisch nodules [17]. From the histopathological standpoint, as 85% of our patients had Lisch nodules, one may think presence of Lisch nodules as an essential contributor to thicker iris profile in the present study. However, further research with histopathological observations is still required to better understand the impact of iris changes on ICA morphology in NF1 cases.

One important observation in our study was significantly smaller SCD in NF1 children than in controls. It has been known that glaucoma may develop during infancy, childhood and adolescence in NF1 cases with a reported incidence of 1/300 [18–20]. One of the proposed mechanisms for pathogenesis of glaucoma in NF1 is the presence of structural alterations in the ICA [3, 18–21]. In line with this assumption, investigators have elaborated goniotrabecular malformations in these patients. For that purpose, Quaranta et al. [4] have performed gonioscopy in juvenile NF1 cases and observed characteristic findings like very narrow ciliary body band indicating poorly developed ICA. Furthermore, Edward et al. [18] have studied clinicopathologic features of NF1 patients aged from birth to 13 years who developed glaucoma in the presence of ectropion uvea. In that study, authors have observed absence of SC in some histopathological specimens. Actually, these observations partly support our finding regarding significantly smaller SCD in NF1 children than in controls in terms of dysgenetic development of the ICA. Although changes in angle morphology observed in NF1 children in our study might suggest a distinct developmental trajectory, they may also be early manifestation of abnormalities seen in adult NF1 individuals. In other words, ICA abnormalities may have a chance to be congenital, progressive, or specific to a certain developmental phase. Thus, ICA narrowing in NF1 children might be static or is likely to worsen with age, potentially increasing the risk of angle-related pathology. It seems that we still need further clinical evidence exploring this issue. Association between SC developmental abnormalities and glaucoma development have been well studied and demonstrated [22, 23]. But, we still do not have any longitudinal data to establish a causal link between our findings and future glaucoma risk in NF1 children. In consideration of close relationship between SC and aqueous humour outflow resistance [24, 25], one may think potential integration of these measurements into clinical screening for paediatric NF1 patients with the use of SD-OCT to assess the possible risk of glaucoma development during the disease course. Furthermore, the observation of significant association of increased IT and narrower SCD in our study may offer a potential influence of iris morphology on SC lumen changes. As we could not give an underlying reason for this outcome, we believe this result necessitates further elaboration.

Over recent years, SSL has gained attention for playing a possible role in glaucoma development [26]. Investigators have reported shorter SSL in open angle glaucoma patients compared to healthy individuals. SSL has also assumed to be a key factor to determine SC lumen alterations, making it to be a potential marker for glaucoma assessment [11]. We also measured SSL in our study based on the measurement methods that have already been used previously [11, 26]. But, we could not identify a difference of SSL between NF1 cases and healthy controls following both SSL measurement modalities. However, one may think the close association between SC and SS, hence future alterations in SSL may be expected in the presence of smaller SCD in NF1 children. Therefore, we believe that SSL analysis needs further investigation in this specific patient population.

Investigators have also shown an association between SC lumen alterations and IOP changes. IOP elevation significantly reduced SC cross-sectional area in healthy eyes [27, 28]. One may think that a change in IOP would have an impact on SCD resulting in decreased SCD in NF1 group in our study. However, similar IOP levels between the two groups might avoid this possible confounder effect in the present study.

Identification of SC during AS-OCT examinations might be difficult especially in paediatric population. But, modern-day SD-OCT technology allows us to detect and properly identify SC, as already described by previous studies in both adult and paediatric subjects [29–31]. With the use of SD-OCT device in our study, we were able to identify SC in 20 of 23 (86.9%) children and in 33 of 35 (94.2%) children, in NF1 and control groups, respectively. These results were consistent with those of reported in SD-OCT studies regarding SC assessment in healthy individuals or those with glaucoma [6, 32]. In a study analysing SC measurements in a large cohort of healthy children, authors have found a lower rate of SC identification in children those under 7 years of age [33]. This finding may also account for relatively higher identification rate of SC in the present study, such that no children were present under 7 years of age during our study period. Furthermore, previous studies evaluating the ICA structure with OCT in children have reported good reproducibility of measurements [14, 33]. Consistently, our intra-observer ICC values indicate excellent agreement for all measured SD-OCT variables. However, manual identification of SC still remains a limitation. Also, automated analysis algorithms are essential clinical applications exploiting the diagnostic accuracy of OCT in order to improve reproducibility of the data [34]. Hence, manual SC identification, lack of interrater reliability assessment and analysis without an automated segmentation software could be considered as potential drawbacks of this study.

In conclusion, we found narrower ACD and decreased values of ACA, AOD and TISA with thicker iris morphology in children with NF1 when compared to those in normal healthy children. SSL measurements were found to be similar between the groups. Apart from narrower ICA morphology, we generated a novel finding that SCD was significantly smaller in children with NF1 than in controls. We believe this result would have a potential clinical relevance. Furthermore, based upon the correlation outcomes in our study, increased IT in NF1 children may also offer a possible influence of iris morphology on narrower ICA parameters and SCD. We believe that further studies are warranted in order to validate the results of the current work and to better ascertain the ICA morphological status in NF1 children.

Supplemental material is available at Eye’s website.

## Summary

### What was known before:

- Narrower iridocorneal angle (ICA) structure was previously shown with the use of optical coherence tomography (OCT) device in adult Neurofibromatosis type 1 (NF1) patients. No information is present regarding in vivo assessment of ICA morphology in paediatric NF1 cases.

### What this study adds:

- First infant population of NF1 evaluated with OCT for potential ICA alterations. We demonstrated decreased Schlemm’s canal diameter, angle opening distance, trabecular-iris space area and increased iris thickness in NF1 children compared to healthy control subjects by using spectral domain OCT imaging.

## Supplementary information

Supplemental Material

## Data Availability

The datasets generated during and analysed during the current study are available from the corresponding author on reasonable request.
