# Supplementary material for: In vivo analysis of iridocorneal angle parameters with spectral-domain optical coherence tomography in children with Neurofibromatosis type 1
Source: Eye (Lond). 2025 May 14;39(11):2217–22. doi: 10.1038/s41433-025-03840-z (PMC12274460; doi:10.1038/s41433-025-03840-z)
Supplement: Supplementary file 1 — Supplemental Material [file 41433_2025_3840_MOESM1_ESM.docx]

Supplemental Material

**Reproducibility of measurements of the iridocorneal angle parameters made by Spectral domain optical coherence tomography**

| Intra-observer  N = 53 | Mean | ICC | 95% CI |
| --- | --- | --- | --- |
| SCD (µm)  Session 1  Session 2  Session 3 | 290.17  281.13  289.19 | 0.996 | 0.994 – 0.998 |
| ACA (degrees)  Session 1  Session 2  Session 3 | 38.61  38.43  38.58 | 0.999 | 0.999 – 0.999 |
| AOD500 (µm)  Session 1  Session 2  Session 3 | 526.97  516.34  523.09 | 0.999 | 0.999 – 0.999 |
| AOD750 (µm)  Session 1  Session 2  Session 3 | 705.17  694.79  704.77 | 0.996 | 0.994 – 0.998 |
| TISA500 (mm^2^)  Session 1  Session 2  Session 3 | 0.181  0.175  0.176 | 0.975 | 0.973 – 0.981 |
| TISA750 (mm^2^)  Session 1  Session 2  Session 3 | 0.339  0.337  0.338 | 0.997 | 0.995 – 0.998 |
| SSL-1 (µm)  Session 1  Session 2  Session 3 | 189.94  186.13  190.38 | 0.979 | 0.968 – 0.987 |
| SSL-2 (µm)  Session 1  Session 2  Session 3 | 157.19  153.06  157.12 | 0.983 | 0.973 – 0.989 |
| IT-1 (µm)  Session 1  Session 2  Session 3 | 342.36  331.75  340.83 | 0.998 | 0.996 – 0.999 |
| IT-2 (µm)  Session 1  Session 2  Session 3 | 303.19  295.26  303.43 | 0.997 | 0.996 – 0.998 |
| IT-3 (µm)  Session 1  Session 2  Session 3 | 275.89  267.32  274.33 | 0.997 | 0.995 – 0.998 |

SCD : Schlemm canal diameter; ACA : anterior chamber angle; AOD : angle opening distance; TISA : trabecular-iris space area; SSL : scleral spur length; IT : iris thickness
